# Stereotactic body radiotherapy for recurrent hemoptysis due to chronic pulmonary aspergillosis: a case report and systematic review of the literature

**DOI:** 10.1007/s00066-022-02013-1

**Published:** 2022-10-20

**Authors:** Alexander Koch, Daniel H. Schanne, Gunar Günther, Daniel M. Aebersold, Olgun Elicin

**Affiliations:** 1 Department of Radiation Oncology, Inselspital, Bern University Hospital, University of Bern, Bern, Switzerland; 2 Department of Pulmonary Medicine and Allergology, Inselspital, Bern University Hospital, University of Bern, Bern, Switzerland; 3 Department of Medical Sciences, UNAM School of Medicine, University of Namibia, Windhoek, Namibia

**Keywords:** CPA, Aspergilloma, Mycetoma, Post-tuberculosis lung disease, SBRT

## Abstract

**Purpose:**

Chronic pulmonary aspergillosis (CPA) can manifest as fungus balls in preexisting cavities of lung parenchyma and recurrent hemoptysis is among the most frequent complications. Radiotherapy can be considered for treatment-refractory aspergilloma and severe hemoptysis. To the best of our knowledge, we present the first application of stereotactic body radiotherapy (SBRT) for a pulmonary aspergilloma in a patient with limited functional lung capacity. The topic was further expanded on with a systematic review of the literature addressing the implementation of radiotherapy in CPA patients.

**Case report:**

A 52-year-old man presented with recurring and treatment-refractory hemoptysis caused by chronic cavitary aspergillosis localized in the left lower lobe. We applied SBRT on two consecutive days with a total dose of 16 Gy. Hemoptysis frequency decreased to a clinically insignificant level.

**Systematic review:**

We performed a systematic search of the literature in line with the PRISMA statement. The initial PubMed search resulted in 230 articles, of which 9 were included.

**Results:**

The available literature contained 35 patients with CPA who received radiotherapy. Dose fractionation usually ranged from 2 to 4 Gy per fraction, applied almost exclusively in conventional two-dimensional (2D) techniques. There is no report of SBRT usage in such a scenario. Most cases report a positive treatment response after irradiation.

**Conclusion:**

The presented case demonstrates long-term clinical stability after SBRT for recurrent hemoptysis due to pulmonary aspergilloma. The systematic literature search revealed that concept definition is still uncertain, and further work is necessary to establish radiotherapy in clinical practice.

## Introduction

Pulmonary aspergillosis can be subdivided into allergic bronchopulmonary aspergillosis (ABPA), invasive pulmonary aspergillosis (IPA), and chronic pulmonary aspergillosis (CPA) [1]. While ABPA is characterized by inflammatory immune-mediated pulmonary processes caused by exposure to *Aspergillus* antigens, IPA is an acute and severe fungal infection of the lung, mostly affecting heavily immunocompromised patients treated with chemotherapy and/or immunosuppressive therapies for conditions such as hematologic malignancies, solid tumors, or organ transplantations [1, 2]. Invasive growth of the *Aspergillus* hyphae can even lead to hematogenous dissemination to distant organs including the central nervous system [1]. In contrast, CPA usually affects immunocompetent individuals with underlying pulmonary diseases. Risk factors include pulmonary tuberculosis (TB), which is the most frequent predisposing factor, but also other diseases causing pulmonary fibrosis and cavity formation such as treated lung cancer, chronic obstructive pulmonary disease (COPD), or fibrocystic sarcoidosis [3, 4]. CPA itself comprises different forms of long-term fungal infections of the lung, varying in clinical manifestation and severity. Chronic cavitary pulmonary aspergillosis (CCPA) and chronic fibrosing pulmonary aspergillosis (CFPA)—its terminal evolution if left untreated—are the most frequent entities. CPA is most commonly caused by *Aspergillus fumigatus*. After colonization of pre-existing pulmonary cavities, it usually presents with aspergillomas, which are composed of fungal hyphae, inflammatory cells, extracellular matrix, and necrotic material [3, 5]. Clinically, CPA manifests with chronic cough, breathlessness, chest pain, fever, weight loss, and hemoptysis. The latter occurs in more than 60% of patients [6]. For diagnosis of CPA, symptoms have to persist for at least 3 months. Besides that, diagnosis of CPA involves microbiological or serological evidence of an *Aspergillus* infection and exclusion of other explanations and suspicious findings in thoracic imaging [3]. Gold standard imaging is CT angiography to precisely localize the aspergilloma and detect possible infiltration of the surrounding vasculature. The affected cavities can vary in wall thickness and contain one or even more fungus balls, often surrounded by a characteristic crescent of air [3]. Surgical resection is considered the preferred and a potentially curative option for symptomatic patients with sufficient respiratory reserve and unilateral or localized manifestations [3, 4, 7]. Additionally, surgery should always be considered in cases of severe hemoptysis [3]. Additionally, long-term antifungal pharmacotherapy with azole derivates or amphotericin B is also recommended for patients with CPA to reduce respiratory symptoms and prevent disease progression [7, 8]. Antifungal medication can also be directly administered into a symptomatic aspergilloma cavity via CT-guided percutaneous or endobronchial instillation [4]. In refractory cases or massive hemoptysis, which occasionally can become life-threatening, bronchial artery embolization (BAE) is an option to stop the bleeding in patients unfit for surgery. However, the recurrence rate of hemoptysis in patients with CPA after BAE is more than 50% [9]. Radiotherapy, on the other hand, is not yet mentioned in the latest multidisciplinary consensus guidelines [7] and currently only considered an option for treatment-refractory aspergilloma and extensive hemoptysis by a small number of clinicians according to the screened literature. To the best of our knowledge, we present the first application of stereotactic body radiotherapy (SBRT) for a pulmonary aspergilloma, delivered with a robotic technique in a patient with limited functional lung capacity. The topic is further expanded on with a systematic review of the literature.

## Methods

### Case report

**Fig. 1:**
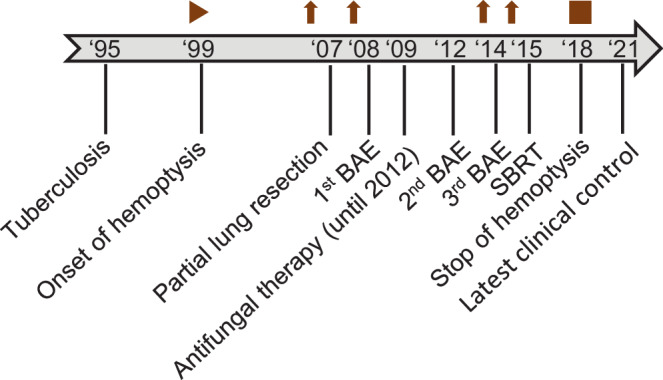
Timeline of important diagnostic and therapeutic steps. The upper part graphically shows onset and end of hemoptysis. The *upward arrows* indicate the most severe episodes of hemoptysis measured by bronchoscopic findings and patient-reported frequency and quantity. *BAE* bronchial artery embolization*, CT* computed tomography*, SBRT* stereotactic body radiotherapy

**Fig. 2:**
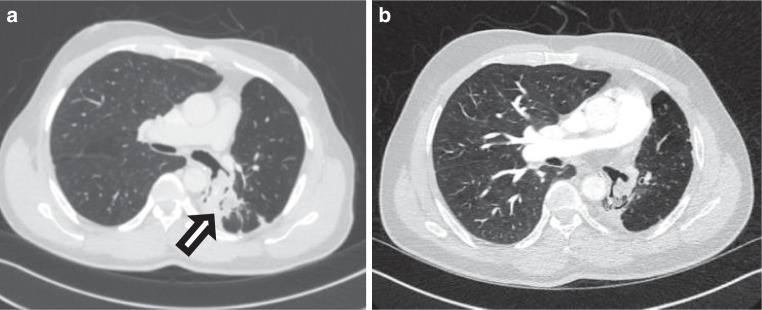
Pre- and postradiotherapy (RT) imaging. **a** Pre-RT thoracic CT scan 8 years after partial surgical resection of the left inferior lobe. The aspergilloma is localized adjacent to the great pulmonary vessels, showing the characteristic radiographic feature of the “air-crescent sign” (*white arrow*). **b** Aspergilloma decreased in size at 3‑year follow-up and no further interventions were necessary. After mild episodes of hemoptysis initially, no further episodes have been reported since then. *CT* computed tomography*, RT* radiotherapy

**Fig. 3:**
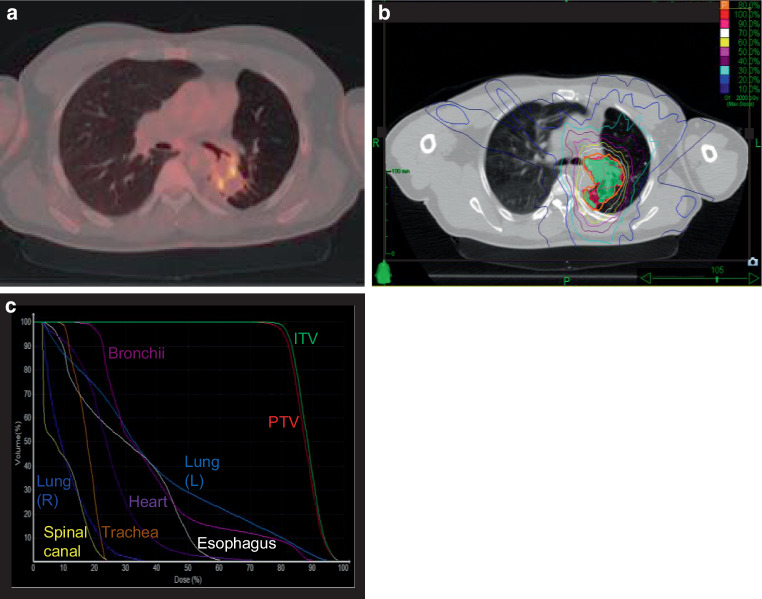
Treatment planning and modalities including dose–volume histogram (DVH). **a** Treatment planning included an ^18^F‑FDG-PET/CT scan to precisely localize the fungal manifestation and the primarily inflamed and eroded vascular lining of the cavity, most likely causing the bleeding. **b** 16 Gy was applied in two fractions of 8 Gy on consecutive days with a robotic arm-mounted linear accelerator. Dose was prescribed to the 80% isodose line. PTV (*red*) was generated from an ITV (*green*) by adding a 2-mm margin. The ITV accounts for motion of the target lesion during respiration registered by a 4D planning CT. The PTV on the other hand accounts for setup and planning uncertainties. **c** The DVH represents the exact radiation dose (shown as percentage of the prescribed dose) delivered to the target volumes and organs at risk (OAR; also shown as percentage of total volume). *CT* computed tomography*, CTV* clinical target volume*, FDG* fluorodeoxyglucose*, ITV* internal target volume*, PET* positron emission tomography*, PTV* planning target volume*, RT* radiotherapy

In 2015, a then 52-year-old man was referred to our radiation oncology department for evaluation of hemostatic treatment. He presented with chest pain, dyspnea, fatigue, loss of appetite, weight loss, recurrent respiratory infections, and episodes of significant hemoptysis. Medical history included pulmonary TB in 1995 (Fig. 1), which was adequately treated with antitubercular medications for 6 months, resulting in a residual cavity in the left lower lobe. Hemoptysis first occurred a couple of years after initial TB treatment and symptoms had become aggravated since then. In 2007, the patient presented with a new episode of hemoptysis. His medical history and a CT scan of the chest lead to the differential diagnosis of pulmonary aspergillosis. The patient was hemodynamically stable and a hemoglobin of 155 g/L (reference value 135–168 g/L) was measured. Since bronchoscopy confirmed endobronchial bleeding from the segments where the lesion was located, partial resection of the left inferior lobe was performed in the same year. Aspergilloma was confirmed by histopathological examination of the resected lung tissue. A post-interventional CT scan of the chest, however, showed persistence of the aspergilloma. In addition, persistence was suggested microbiologically by positive *Aspergillus *cultures from bronchoalveolar lavage fluid and sputum at different timepoints. In the following years, three attempts at endovascular embolization of pulmonary arteries, the first in 2008, were performed due to recurring hemoptysis, without achieving lasting hemostasis. Therefore, long-term antifungal therapy with itraconazole was initiated in 2009 and continued for 2.5 years. In addition to the aspergillosis, the patient was diagnosed with a pulmonary actinomycosis in 2015, a rare opportunistic bacterial infection of the lung. *Actinomyces* were also isolated from a bronchoscopic biopsy in the left lower lobe. Although *Aspergillus* cultures and *Aspergillus* precipitin could not reaffirm aspergilloma persistence at that time, these results did not definitely rule out a concomitant fungal and bacterial infection. The patient’s pulmonary function was further reduced after suffering multiple pulmonary emboli. The last complete pulmonary function testing prior to radiotherapy was performed in 2013. It revealed a vital capacity (VC) of 2.15 L (44% of reference value), forced expiratory volume in one second (FEV_1_) of 1.55 L (41%), and a diffusing capacity of the lungs for carbon monoxide (DLCO) of 7.4 mmol/min/kPa (71%), so that the patient was considered unfit for further surgical interventions. During the first consultation in our clinic, the patient reported recurrent episodes of hemoptysis causing blood loss of more than 100 ml per day. A CT of the chest showed a persistent, spiculated, partly cavernous lesion in the left lower lobe with a diameter of approximately 5 cm (Fig. 2a). Treatment planning also included an ^18^F‑FDG-PET/CT scan to localize the fungal manifestation and identified the metabolically active inflamed vascular lining of the cavity as the most likely cause of bleeding (Fig. 3a). SBRT with a total dose of 16 Gy was applied in two fractions of 8 Gy on consecutive days with a robotic arm-mounted linear accelerator equipped with an iris collimator (CyberKnife®, Accuray Inc., Sunnyvale, CA, USA; Fig. 3b). Dose was prescribed to the 80% isodose line and the ray-tracing algorithm was used for dose calculation. After acquisition of a 4D-planning CT to account for respiratory motion, the planning target volume (PTV) was generated from an internal target volume (ITV), adding a 2-mm margin. Despite an irregularly shaped PTV, the chosen irradiation technique allowed us to achieve a conformal dose distribution of the target volume and tolerable doses for relevant organs at risk (OAR) as shown in the dose–volume histogram (DVH; Fig. 3c). The treatment was well tolerated, and no side effects were reported by the patient. During the 6 years of follow-up at our department, the patient has reported a significant decrease in hemoptysis frequency and volume, and no new long-term medication or invasive treatments have been necessary since then. When he presented at the hospital’s emergency unit with dyspnea and small-volume hemoptysis in 2016, there were no signs of an active or older bleeding evident in CT or bronchoscopy. Hemoglobin level remained stable at around 140 g/L over the years and fell below 120 g/L only once during an episode of community-acquired pneumonia in 2016 not accompanied by hemoptysis. Regular CT scans of the chest confirmed a stable size of the pulmonary lesion after an initial pseudoprogression, which is often observed after SBRT for large target volumes (Fig. 2b; [10]).

## Systematic review

### Search strategy and data collection

We performed a systematic search of the literature to find any reported cases of radiotherapy applied to patients with chronic pulmonary aspergillosis. Due to the limited number of reports, we also included case reports of individual patients. First, we conducted a PubMed search for reports including at least one of the possible combinations of the terms [“mycetoma” or “fung*” or “aspergill*”] and [“lung” or “haemopt*” or “bronchopulmonary” or “pulmonary”] and [“irradiat*” or “radiotherapy” or “radio-therapy” or “sbrt”] in the title or abstract without any limitations regarding the date of publication. Additionally, we performed a cross-reference search with Scopus and screened the databases of both the American Society for Radiation Oncology (ASTRO) and the European Society for Radiation Oncology (ESTRO) for relevant congress abstracts. The literature search was performed in line with the Preferred Reporting Items for Systematic Reviews and Meta-Analyses (PRISMA) statement, meaning that findings were evaluated by two persons separately [11].

## Results

**Fig. 4:**
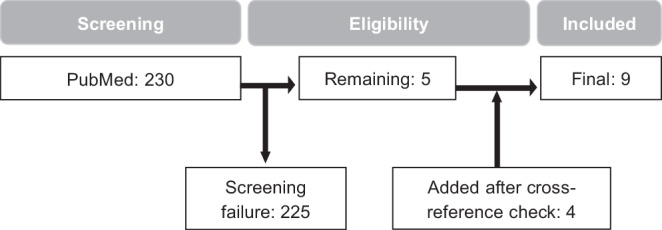
Flowchart of article selection process in line with the Preferred Reporting Items for Systematic Reviews and Meta-Analyses (PRISMA) statement

The initial PubMed search delivered 230 results, of which only 5 fulfilled the thematic criteria. Four articles were manually added after cross-reference checking (Fig. 4). One Spanish article was also included after translation [12]. The final nine articles included 35 patients (Table 1), 21 of them reported in the largest case series so far by Sapienza et al. [13]. Where reported, radiotherapy was delivered in a 2D conventional technique, with opposed fields in most cases. Application of a 3D conformal technique with parallel opposed-oblique fields was only reported for one patient [14]. The prescribed doses ranged from 2 to 4 Gy per fraction, with total doses between 7 and 30 Gy (up to 34 Gy in case of recurrence) [12–20]. Weekly fractions of 3.5 Gy up to varying total doses have been reported by multiple authors [14, 18, 20]. Mean follow-up period across all reports was 4 years and 5 months. None of the studies reported any significant acute or late toxicities with exception of mild cough [13]. Sapienza et al. also reported an improvement of performance status in all patients 30 days after treatment. Almost all patients showed a lasting treatment response, either identified by a reduction of *Aspergillus* precipitin, disappearance of hemoptysis, or a decrease in aspergilloma size in chest imaging. Sapienza et al. calculated a 5-year local control rate of 82% [13]. One patient needed an increased dose of 34 Gy due to persistent hemoptysis after 20 Gy had initially been applied. Another patient of the same cohort was treated with a second course of 20 Gy after recurrence of bleeding [13]. As far as reported, only three of 35 patients died as a direct consequence of recurrent hemoptysis during the reported follow-up periods [13, 14].

**Table 1:** Overview of reported cases applying radiotherapy to patients with pulmonary aspergillosis

First author (year of publication)	Number of patients	Indication	RT-technique	Prescription (total dose/single dose/schedule)	Follow-up	Outcome
Shneerson (1980) [15]	1	Hemoptysis due to aspergilloma	2D conventional; single anterior field initially/parallel opposed fields for relapse	Initially: 20 Gy/4 Gy/7 days Relapse after 8 weeks: additional 10 Gy/2 Gy/7 days	8 months	No further bleeding during follow-up and stable size of aspergilloma
Sahoo (1989) [16]	3	Palliative RT for bronchogenic carcinoma in patients with positive *Aspergillus* antibodies	NR	30 Gy^a^/NR/6 weeks	NR	Disappearance of *Aspergillus* antibodies/seroconversion
Sahoo (1990) [17]	1	Palliative RT for bronchogenic carcinoma in patient with concurrent TB and positive *Aspergillus* antibodies	NR	30 Gy^a^/NR/NR with concomitant antituberculosis chemotherapy	5 months	Disappearance of *Aspergillus *antibodies/seroconversion
Fernandez Vazquez (1994) [12]	1	Hemoptysis due to aspergilloma with underlying TB	NR	30 Gy/NR/NR	NR	No further bleeding reported
Falkson (2002) [18]	5	Hemoptysis due to aspergilloma	2D conventional; anterior–posterior parallel opposed fields with 1.0 cm margin	7–14 Gy/3.5 Gy/once weekly, one additional fraction after hemoptysis stopped	6 months	No further bleeding during follow-up
Glover (2007) [19]	1	Hemoptysis due to aspergilloma with underlying small vessel vasculitis	NR	8 Gy/4 Gy/10 days apart	6 months	No further bleeding during follow-up and radiographic resolution of aspergilloma
Samuelian (2011) [20]	1	Hemoptysis due to aspergilloma	2D conventional; anterior–posterior parallel opposed fields with 1.5 cm margin	28 Gy/3.5 Gy/once weekly	4 years	Hemoptysis resolved after 8 weeks; only clinically insignificant hemoptysis during follow-up; aspergilloma decreased in size
Sapienza (2015) [13]	21	Hemoptysis due to aspergilloma with underlying TB	2D conventional; parallel opposed fields with 1.0–2.0 cm margins	Initially: 20 Gy/2 Gy/daily Persistence (one patient): additional 14 Gy/2 Gy/daily Recurrence (one patient): additional 20 Gy/2 Gy/daily	Mean 73.5 months (1.3–281 months)	5‑year local control: 82% 5‑year overall survival: 59% Two patients died due to recurrence of hemoptysis
Bastin (2020) [14]	1	Hemoptysis due to aspergilloma	3D conformal; opposed-oblique fields with 1 cm CTV and 1 cm PTV margin	7 Gy/3.5 Gy/once weekly	> 7 months (plus several months, not specified)	Patient died due to recurrence of hemoptysis
Current case	1	Hemoptysis due to aspergilloma	SBRT	16 Gy/8 Gy/daily	6 years	Decrease in hemoptysis frequency with only clinically insignificant hemoptysis. No further bleeding in last 3 years

*CTV* clinical target volume, *NR* not reported, *PTV* planning target volume, *RT* radiotherapy, *SBRT* stereotactic body radiotherapy, *TB* tuberculosis

^a^The original reports by Sahoo et al. both mentioned total doses of 3000 Gy, which we assumed to be a typing error regarding the frequent use of cGy as the preferred unit of absorbed doses at that time.

## Discussion

We present a first case of a CPA patient with recurrent hemoptysis despite previous surgery, bronchial artery embolization, and antifungal treatment, whose symptoms were finally controlled successfully by SBRT. As the literature contains only narrative reviews [4, 20] about radiotherapeutic treatment of CPA, we also performed a systematic review.

Hemoptysis is the most frequent and possibly life-threatening complication of CPA. Various treatment approaches have been reported aimed at controlling hemoptysis and disease progression in CPA. An estimated 3 million patients are affected by CPA worldwide. Approximately 20% of patients with post-tuberculosis cavities in the lung [21, 22] suffer from CPA. Therefore, CPA can be regarded as a global health burden, although detailed data of morbidity and mortality barely exist. In this context, it is possible that the utilization of radiotherapy to control hemoptysis in CPA is underreported in the literature.

Radiotherapy is already established as an efficient and well-tolerated option to control bleeding of tumors [23]. Hemostatic radiotherapy is thought to work by inducing edema, endothelial damage causing capillary necrosis, formation of thrombosis, and both vascular and perivascular fibrosis [4, 24]. It is currently not well established whether radiotherapy may additionally have a direct fungicidal effect. *Aspergillus* species can be inactivated by ultraviolet light and visible light irradiation of certain wavelengths can alter their metabolism, growth, and toxin production [25, 26]. A dose–response relationship has been reported for gamma rays. Doses of 10 and 50 Gy increased the in vitro growth of different fungi species, not including *Aspergillus fumigatus*, whereas higher doses of 1000 and 5000 Gy inactivated protein and polysaccharide synthesis [27]. Agricultural studies also show that gamma-irradiation with doses in the magnitude of kGy can be used for effectively decreasing fungal contamination [28, 29]. This might indicate that the lower doses usually applied in patients may not have a direct antifungal effect. However, fungal radioresistance varies greatly between species and the in vivo effect of radiation-induced tissue reaction on growth and metabolism of *Aspergillus fumigatus* has not been widely studied.

Consequently, the ideal dose fractionation for hemostatic radiotherapy is still discussed controversially. Depending on tumor entity, anatomic localization, and severity of bleeding, fractionation schemes are typically in the range of 10 × 2 Gy, 4–13 × 3 Gy, 3–6 × 4 Gy, or 4–5 × 5 Gy [23]. However, short-course hypofractionated schemes such as 1 × 8 Gy were shown to achieve equal bleeding control of approximately 90%, with lower risks of treatment interruption, incompliance, and hospitalization [30]. A single fraction of 8 Gy also emerged as the preferred scheme for hemoptysis in a large survey among Dutch radiation oncologists [31]. The reported fractionation schemes used for patients with hemoptysis caused by CPA may have been guided by the established and conservative hemostatic schemes. In contrast, we applied an escalated dose of 2 × 8 Gy on consecutive days in the reported case with great success and no reported side effects. This was chosen in consideration of the younger patient age and the fact that this was not a palliative cancer setting. In general, hypofractionation with SBRT also has logistic advantages and can spare a patient with limited respiratory reserve multiple trips to the hospital. The stereotactic technique and the localization of the pulmonary lesion further allowed us to avoid excess doses to organs at risk (Fig. 3c). However, precise treatment planning, dose calculation and treatment delivery is a necessity when performing SBRT to larger targets to avoid excess toxicity, especially in cases of centrally located thoracic target volumes. Some reports indicate an increased risk of pneumonitis, bronchopulmonary hemorrhage, fistulation, or neuropathy [32, 33]. Radiation-induced edema might also cause transient dyspnea. However, these studies applied considerably higher doses of at least 8 × 5 Gy or even 7 × 8 Gy in an oncologic setting. On the other hand, for more peripherally located thoracic tumors, even SBRT with one to three fractions with higher biologically equivalent doses did not result in an increase in major toxicity [34]. We therefore did not expect major side effects for the chosen fractionation in our case. Moreover, there is no plausible radiobiological explanation not to apply the dose hypofractionated or as a single fraction in this setting, because there is no known alpha/beta ratio difference between the target volume (i.e. fungus-infested tissue) and the tissue in its vicinity to exploit in order to protect the healthy tissue.

Notably, young age, blood-tinged sputum, and thick wall cavities have been identified as independent risk factors for severe hemoptysis in patients with aspergilloma [35]. The fact that the configuration of the cavity wall but not the initial size of the fungus ball is an independent prognostic marker might also speak for the beneficial use of additional ^18^F‑FDG-PET/CT imaging to identify particularly eroded and inflamed parts of this critical area. PET imaging has already become an essential part of the daily routine in radiation oncology [36]. In fact, it is also implemented in the management of invasive fungal infections for evaluating disease activity, expansion, and treatment response to antifungal agents [37, 38]. Regarding other radiologic modalities, respiratory-gated thoracic MRI imaging may also offer potential benefits for treatment planning by helping to identify eroded and affected parts of the aspergilloma wall with increased spatial resolution.

Comparing hemostatic radiotherapy to BAE as the current standard of care for hemoptysis in patients unfit for surgical interventions, both treatment modalities show great efficacy. Independent of the etiology of bleeding, BAE is particularly efficient for immediate hemostasis, with complete cessation of massive hemoptysis within the first 24 h in 85–96% of patients. However, rare but severe complications such as strokes, transient ischemic attacks, and transient quadriplegia are reported in some patients following BAE [39, 40]. Therefore, while BAE is the standard for acute and life-threatening cases of hemoptysis, hemostatic radiotherapy may be suitable for less severe scenarios and consolidation after initial BAE to prevent recurrence and the necessity of multiple interventions.

One obvious limitation of the present work is the limited number of reported cases. Given the favorable benefit–risk profile, larger studies of hemostatic radiotherapy in patients with aspergillosis appear viable. The CPAnet database is a prospective clinical register which is currently trying to improve the evidence base for management of CPA [41]. The direct antifungal effect of ionizing radiation, ideal dose and fractionation, and combination with BAE and/or antifungal medication would be of particular interest. Unfortunately, the highest burden of CPA is likely in low- and middle-income countries, where access to diagnostics and treatments like BAE, robotic linear accelerators, and PET/CT scanners may be limited, restricting treatment options for refractory cases of CPA with hemoptysis. However, while PET imaging may indeed be beneficial for target delineation, it is not indispensable, and SBRT can also be applied with most conventional gantry linear accelerators with sufficient precision in such scenarios.

## Conclusion

We demonstrate the feasibility of long-term control of recurrent hemoptysis after SBRT due to CPA. SBRT ensured conformal dose delivery and acceptable radiation exposure of OARs for a large and irregularly shaped target volume. Further studies are necessary to identify the ideal dose fractionation and the most effective sequence when different conservative treatment modalities such as radiotherapy, BAE, and antifungal medication are combined. However, these are difficult to perform due to the relative scarcity of CPA cases in settings with adequate resources. An intensified interdisciplinary collaboration between pneumologists, thoracic surgeons, infectious disease specialists, and radiation oncologists, facilitated by case registries such as CPAnet, seems to be a good approach to improve treatment outcomes and gaining further insight into the optimal management of CPA.

## Abbreviations

ABPA: Allergic bronchopulmonary aspergillosis
BAE: Bronchial artery embolization
CCPA: Chronic cavitary pulmonary aspergillosis
CFPA: Chronic fibrosing pulmonary aspergillosis
COPD: Chronic obstructive pulmonary disease
CPA: Chronic pulmonary aspergillosis
CT: Computed tomography
CTV: Clinical target volume
DLCO: Diffusing capacity of the lungs for carbon monoxide
DVH: Dose–volume histogram
^18^F-FDG: (18)F‑fluorodesoxyglucose
FEV_1_: Forced expiratory volume in one second
IPA: Invasive pulmonary aspergillosis
ITV: Internal target volume
NR: Not reported
OAR: Organs at risk
PET: Positron-emission tomography
PTLD: Post-tuberculosis lung disease
PTV: Planning target volume
RT: Radiotherapy
SBRT: Stereotactic body radiotherapy
TB: Pulmonary tuberculosis
VC: Vital capacity
